# Silent Speech Decoding Using Spectrogram Features Based on Neuromuscular Activities

**DOI:** 10.3390/brainsci10070442

**Published:** 2020-07-11

**Authors:** You Wang, Ming Zhang, RuMeng Wu, Han Gao, Meng Yang, Zhiyuan Luo, Guang Li

**Affiliations:** 1 State Key Laboratory of Industrial Control Technology, Institute of Cyber Systems and Control, Zhejiang University, Hangzhou 310027, China; king_wy@zju.edu.cn (Y.W.); drystan@zju.edu.cn (M.Z.); 21932107@zju.edu.cn (R.W.); gao_han@zju.edu.cn (H.G.); 2 Department of Computer Science and Technology, School of Mechanical Electronic and Information Engineering, China University of Mining and Technology, Beijing 100083, China; m.yang@cumtb.edu.cn; 3 Computer Learning Research Centre, Royal Holloway, University of London, Egham Hill, Egham, Surrey TW20 0EX, UK; zhiyuan.luo@cs.rhul.ac.uk

**Keywords:** silent speech decoding, neuromuscular signal, spectrogram features, Xception, bidirectional long short-term memory

## Abstract

Silent speech decoding is a novel application of the Brain–Computer Interface (BCI) based on articulatory neuromuscular activities, reducing difficulties in data acquirement and processing. In this paper, spatial features and decoders that can be used to recognize the neuromuscular signals are investigated. Surface electromyography (sEMG) data are recorded from human subjects in mimed speech situations. Specifically, we propose to utilize transfer learning and deep learning methods by transforming the sEMG data into spectrograms that contain abundant information in time and frequency domains and are regarded as channel-interactive. For transfer learning, a pre-trained model of Xception on the large image dataset is used for feature generation. Three deep learning methods, Multi-Layer Perception, Convolutional Neural Network and bidirectional Long Short-Term Memory, are then trained using the extracted features and evaluated for recognizing the articulatory muscles’ movements in our word set. The proposed decoders successfully recognized the silent speech and bidirectional Long Short-Term Memory achieved the best accuracy of 90%, outperforming the other two algorithms. Experimental results demonstrate the validity of spectrogram features and deep learning algorithms.

## 1. Introduction

Research on Brain–Computer Interfaces (BCI) has a long history [1] and has attracted more attention for its extensive potential in the fields of neural engineering, clinical rehabilitation, daily communication and many other possible applications [2,3,4]. A typical non-invasive BCI uses electroencephalography (EEG) as it is inexpensive and easy to implement [5]. However, the difficulty in data processing still remains for practical use. One promising approach to address the challenge is the neuromuscular decoding from articulatory muscles [6]. Surface Electromyography (sEMG) captures neuromuscular activities in a non-invasive way like EEG. Besides, it only requires a few channels for signal processing due to the neural pathway from the brain to muscle acting as a primary filter and encoder [7,8,9].

In the accessible area around the face, surface electrodes are placed on articulatory muscles to obtain speech-related sEMG, both in vocal and silent speech [6,10,11,12]. Some other techniques are also used in the silent speech recording. Video and ultrasound imaging can record the movements of visible or invisible speech articulators straightforwardly [13,14]. However, they do not work in purely silent speech without any articulator motion.

The primary use of sEMG for silent speech recognition can date back to the mid-1980s, when Sugie in Japan [15] and Morse in the United States [16] demonstrated that sEMG signals contain speech-related information, respectively. Using simple thresholding techniques, Sugie utilized a three-channel electrode to distinguish five Japanese vowels, verifying that they could run in a pilot real-time system [15]. Later, Morse obtained linguistic information from muscle activities of neck and head, successfully distinguishing two words [16]. In the following years, the word number expanded to ten with an accuracy of 70% [17]. However, when it increased to 17, the accuracy dropped to only 35% [18]. In 2001, Chan reported the work of recognizing 10 English numbers based on sEMG during speech, using a wavelet transform feature set with linear discriminant analysis [19]. Later on, a group of researchers utilized sEMG to identify six commands to control an aircraft [20]. Szu-Chen studied continuous audible speech recognition using sEMG, achieving a 32% error rate by decomposing the signal into different feature spaces in time domain [21]. In 2014, Wand used four polar and two bipolar electrodes to capture sEMG to achieve the best average silent speech error rate at 34.7%, where zero-crossing rate, mean value and signal power were extracted [9]. Early in 2018, Kapur reported a wearable silent speech interface to obtain good accuracy around 90%, using a convolutional neural network [6]. Later, Meltzner demonstrated that silent speech was recognized with high accuracy using vocal speech features on a large data set [22].

Although multiple electrodes lead to multi-channel sEMG, previous studies mostly focus on channel-wise features which are extracted on a channel-by-channel basis, while correlations between channels are ignored. Speech is produced by the synergistic work of vocal system and articulatory neuromuscular activities occur along with these physiological processes [23,24,25]. Even in silent speech, such signals can be recorded and synergistic mechanism exists among the muscles. So, synergistic features from multi-channel sEMG are considered to recognize different words. Xception, originally designed for image classification [26,27,28,29], is utilized to process spectrograms of multichannel sEMG to explore the spatial correlation.

In this paper, multi-channel sEMG of silent speech are recorded. Xception is exploited to extract spatial correlative features. Three deep learning methods, Multi-Layer Perceptron (MLP), Convolutional Neural Network (CNN) and bidirectional Long Short-Term Memory (bLSTM), are evaluated to decode silent speech.

## 2. Silent Speech Data

### 2.1. Capturing Speech-Related sEMG

**Figure 1:**
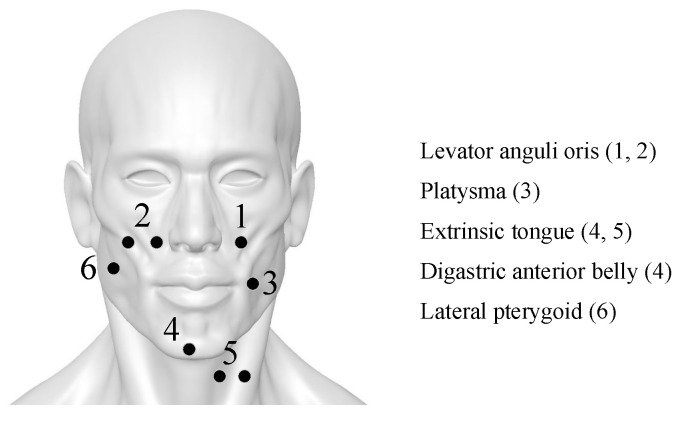
Recording sites around the face and neck. These dedicated positions form an articulator muscular net to decode the silent speech. The sites are cleaned by gel to ensure the impedance is lower than 5 kΩ between electrodes and skin surface.

Studying the relationships between vocalization and articulatory muscles, we select suitable electrode positions around the face [6,8,30,31,32,33], as shown in Figure 1. Channels 2 and 5 are bipolar derivation to improve the common-mode rejection ratio (CMRR) while others are derived unipolarity. Channels 1 and 2 record the levator anguli oris while channel 4 captures both the extrinsic tongue and the digastric anterior belly. Channels 3, 5 and 6 record the platysma, the extrinsic tongue and the lateral pterygoid, respectively. Besides, two reference electrodes are placed on the mastoid behind ears.

There is no articulator motion in silent speech, so the amplitude of sEMG is generally below 1 mV, smaller than normal sEMG. The frequency band of silent speech sEMG is always no more than 300 Hz. In our data recording system, the bandwidth is approximately 5 kHz and a 24-bit analog-to-digital converter (ADC) is used. Two resistor–capacitor (RC) filters, including a direct current (DC) filter and a 5 kHz low-pass filter are exploited to eliminate the DC bias and high-frequency interference, respectively. sEMG data are recorded at a sampling rate of 1000 Hz.

Seven students with normal vision and oral expression skills, having no history of mental illness and neurological diseases, 20 to 25 years old (average 22, four males and three females), are recruited as subjects. The experiment named “BCI research based on transmitted neural signals” has been approved by Ethics and Human and Animal Protection Committee of Zhejiang University (Ethical Approval: ZJUEHAPC2019-CSEA01), and strictly follows the Declaration of Helsinki. All collected data are only used for data analysis and the privacy of the participants are firmly protected.

**Figure 2:**
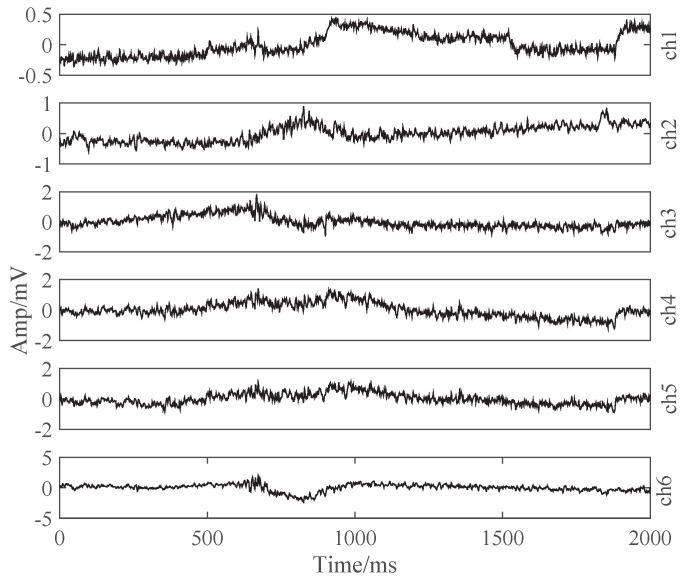
An example of six-channel surface electromyography (sEMG) when imagining to speak ‘decelerate’ in Chinese.

The six-channel sEMG is recorded while the subjects are trained to imagine speaking the labelled words displayed on a computer screen one by one in a defined sequence, which is the meaning of silent speech in this paper. In our experiments, ten Chinese words are selected, including ’噪’, ’1#’, ’2#’, ’前’, ’后’, ’左’, ’右’, ’快’, ’慢’, ’停’, which mean ’null’, ’No.1’, ’No.2’, ’forward’, ’backward’, ’left’, ’right’, ’accelerate’, ’decelerate’, ’stop’ in English, respectively. In total, 69,296 valid samples for the ten words are recorded, and the label distribution is various, as shown in Table 1. Figure 2 illustrates a valid six-channel sEMG example.

### 2.2. Preprocessing

**Figure 3:**
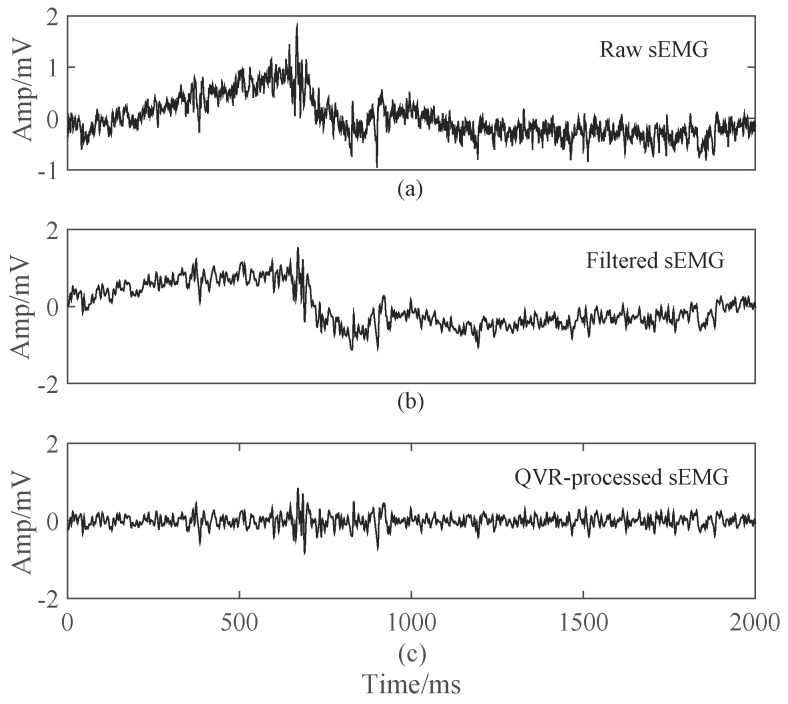
Preprocessing of sEMG. (**a**) An example of raw sEMG, corresponding to channel 2 in Figure 2; (**b**) The sEMG filtered by Butterworth (0.15∼300 Hz) and notch (50 Hz) filters; (**c**) Quadratic Variation Reduction (QVR)-processed sEMG, where the most amplitude change is less than 1 mV.

An 8th order Butterworth bandpass filter (0.15∼300 Hz) was applied to remove the DC bias and high frequency of sEMG. The power frequency of 50 Hz and its harmonics was filtered by a comb notch filter [6,34,35,36]. The filtered sEMG is then obtained, as shown in Figure 3b.

In order to remove the baseline drift, the Quadratic Variation Reduction (QVR) [37] method is applied:

(1) $$z=\left[I-{\left(I+\lambda D^{T}D\right)}^{-1}\right]\tilde{z}$$

where $\tilde{z}$ and z denote the signal before and after using QVR, λ is a constant value (λ=100), I represents the identity matrix and D is a (n−1)×n matrix:

(2) $$D=\left[\begin{array}{ccccc} 1 & -1 & 0 & \cdots & 0\\ 0 & 1 & -1 & \ddots & \vdots \\ \vdots & \ddots & \ddots & \ddots & 0\\ 0 & \cdots & 0 & 1 & -1 \end{array}\right]$$

where *n* is the length of $\tilde{z}$.

In Equation (1), ($I+\lambda D^{T}D$) is a symmetric, positive-definite, tridiagonal matrix, which can be solved efficiently. The effect is shown in Figure 3c where it can be seen that most wander part is removed.

## 3. Processing Methods

**Figure 4:**
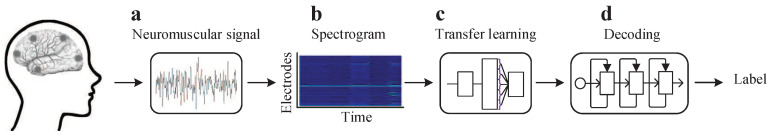
Silent speech decoding. (**a**): The neuromuscular activities are captured by surface electrodes; (**b**): All data are transformed into spectrograms by short-time Fourier transform (STFT); (**c**): Transfer learning method is used to extract features from spectrograms; (**d**): Neural networks decode multi-channel sEMG using the extracted features.

In order to extract time–frequency features effectively, the original six-channel sEMG in the time domain were transformed into the frequency domain, creating a spectrogram which is represented as an image. The state-of-the-art model Xception was selected for extracting image features, which were then decoded by MLP, CNN and bLSTM, respectively. Figure 4 describes the processes to decode sEMG.

### 3.1. Spectrogram Images

The spectrogram of a signal sequence is the visual representation of the magnitude of the time-dependent Fourier Transform (FT) versus time, also known as the short-time Fourier transform (STFT) [38,39,40]. It describes the spectral details in time-frequency domain.

(3) $$\mathrm{Spectrogram}={\left|STFT\left(x\right)\right|}^{2}.$$

**Figure 5:**
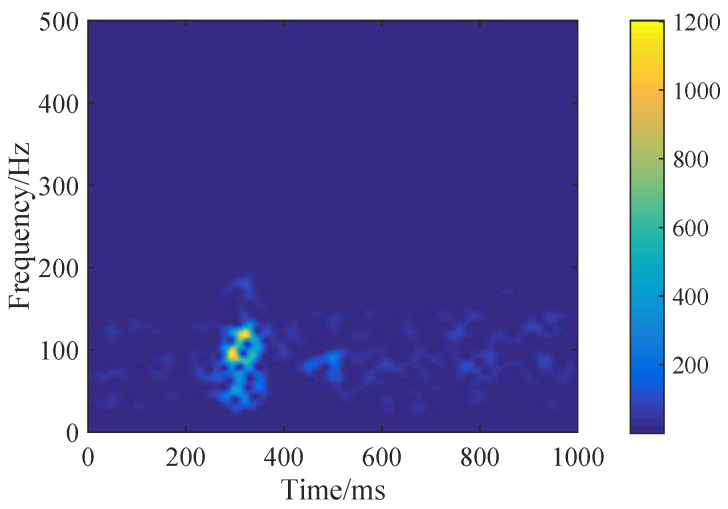
An example of a spectrogram image.

The spectrogram was calculated by Equation (3) [38], where the parameters of [window, window length, sample rate, overlap, FFT length] were specified as [hanning, 512, 1000 Hz, 50%, 64]. An example of a spectrogram image is shown in Figure 5. The images associate with each other, reflecting sEMG spatial relationships in the frequency domain. Inspired by short video streams, the images were treated as a fixed-size video. Then, the silent speech decoding becomes a video classification, explored by deep learning methods.

### 3.2. Feature Extraction

To explore sEMG spatial features, transfer learning with Xception is used. It is a deep learning image classifier using depthwise separable convolution layers with residual connections, which has been pre-trained on large scale images [26]. After input, data using only pointwise convolution (1 × 1 convolution) create separate convolution sizes of 3 × 3 without average pooling, which proceeds in nonoverlapping sections of the output channels to then be fed-forward for concatenation [26,27]. The model demonstrates a strong ability to generalize to images outside the original dataset via transfer learning, such as feature extraction and fine-tuning. Fine-turning is done by training all weights with a smaller learning rate, removing and updating some biased weights from the original network.

The spectrogram images have various shapes and are scaled to 299 × 299. Xception model outputs 1000 features for each image, therefore 1000 × 6 = 6000 features are obtained for one sEMG sample. All samples are processed using Xception to generate a large feature set.

### 3.3. Decoder Design

**Figure 6:**
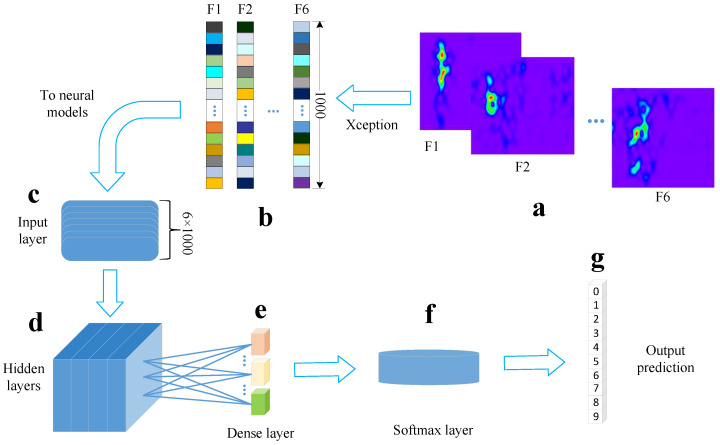
Decoding processes. (**a**) Spectrogram images. (**b**) Feature set extracted by Xception. (**c**) Input layer of neural networks. (**d**) Hidden layers of neural networks. (**e**) Fully connected dense layer. (**f**) Softmax layer as the output layer. (**g**) The predicted labels we obtain from the models.

Three deep learning methods, namely MLP, CNN and bLSTM, are explored using the above feature set. Their structure and parameter details are designed in this section [41,42,43]. Figure 6 illustrates our decoding process, where parts (c)∼(g) represent the common structures and components for the three models, except that different hidden layers and parameters are used in each model.

#### 3.3.1. MLP

Multi-Layer Perceptron (MLP) is a common Artificial Neural Network (ANN). In addition to the input and output layers, there can be multiple hidden layers. MLP can also be thought of as a directed graph consisting of multiple layers, each fully connected to the next layer [44,45].

**Figure 7:**
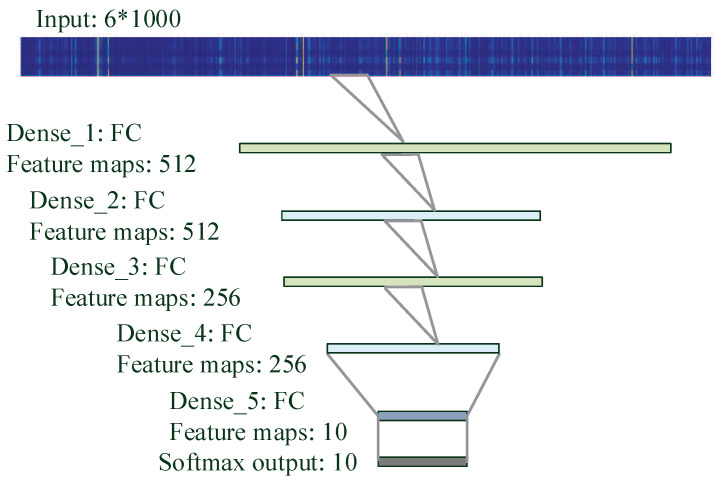
MultiLayer Perceptron (MLP) architecture to decode silent speech. A feature vector goes through the layers and a digital (from 0 to 9) will be output.

Figure 7 illustrates the MLP structure where the ’dense’ layer connects each input unit with each output unit of the layer to learn and update the weights. ’Dropout’ regularization is used to help prevent overfitting as it randomly drops out input units with a fixed rate during parameter tuning [46]. ‘Softmax’ calculates predicted label probabilities at the output layer and then outputs the label with the maximum probability. The loss function defined in this method is cross-entropy loss.

Each hidden layer uses a non-linear activation function to enhance the performance of the neural network and solve the linear inseparable problem. Commonly used activation functions are sigmoid, tanh, and rectified linear unit (ReLU). ReLU is used in the MLP as the derivative of ReLU is always 1 in the positive interval, alleviating the gradient disappearance and gradient explosion problems. In addition, ReLU has a much faster convergence than sigmoid and tanh.

#### 3.3.2. CNN

Convolutional Neural Network (CNN) features with local connections and shared weights, making it very popular and successful in image classification problems. The core operation of CNN is mathematical convolution which consists of filters. The convolution is applied on the input data to produce a feature map. Specifically designed filters can extract features via convolution [47,48,49].

**Figure 8:**
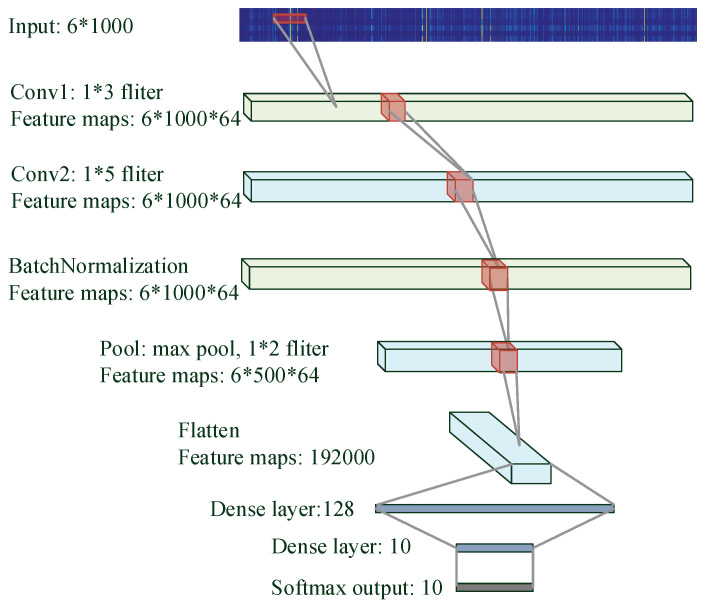
Convolutional Neural Network (CNN) architecture to decode silent speech.

The CNN structure is shown in Figure 8, where two convolutional layers (Conv1 and Conv2) with different filters are used to create specific feature maps. The pooling layer provides downsampling to reduce the size of features and also helps prevent overfitting. Max pooling that calculates the maximum value for each patch is used in our CNN architecture.

In the neural networks, the output of the first layer feeds into the second layer, and the output of the second layer feeds into the third, and so on. When the parameters of a layer change, so does the distribution of inputs to subsequent layers [50], which is described as an internal covariate shift. These shifts in input distribution can be problematic for neural networks, especially deep neural networks that have a large number of layers [51]. Batch normalization, a technique to standardize the inputs to a layer and reduce unwanted shifts to speed up training [52], is used in the CNN model.

#### 3.3.3. bLSTM

Recurrent Neural Network (RNN) is well-known for processing sequence data, and has made many significant accomplishments in natural language processing applications. Unlike MLP and CNN, the output of each hidden layer in RNN are stored as memory and can be considered as another input, by which it allows information to persist [47,49,53]. However, RNNs suffer from short-term memory. If a sequence is long enough, they will have a hard time carrying information from earlier time steps to later ones. During back propagation, the gradient vanishing in RNN is a serious problem when learning long-term dependencies [53]. The gradient shrinks in back propagation and it does not contribute much to learning if it becomes extremely small [54].

LSTM, a special kind of RNN, addresses this issue by considering that both memory and input operations are addition only. As a result, it is capable of learning long-term dependencies [49]. The core concept of LSTM is the cell state and its various gates. The cell state acts as a transport highway that transfers relative information all the way down the sequence chain. LSTM has the ability to remove or add information by gates. There are the forget gate, input gate and output gate to regulate the flow of information inside the LSTM unit and learn which data in a sequence is important to keep or dismiss.

**Figure 9:**
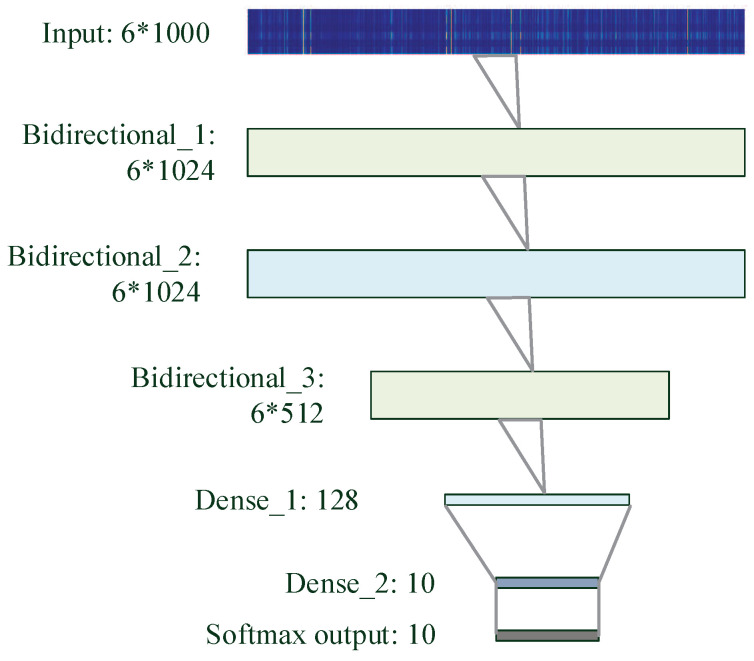
Bidirectional Long Short-Term Memory (bLSTM) architecture to decode silent speech.

bLSTM, including forward LSTM and backward LSTM, captures bidirectional semantic dependencies [44,54]. For six-channel sEMG, bLSTM tends to be a suitable classifier as it can effectively model bidirectional dependencies. Figure 9 shows details of the bLSTM architecture, consisting of three bidirectional layers, two dense layers and one softmax output layer.

## 4. Results

### 4.1. Decoder Optimization

**Figure 10:**
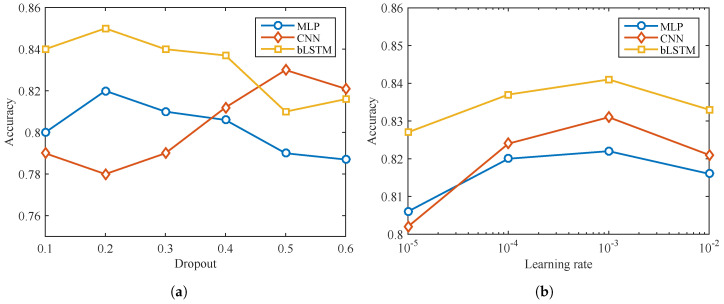
Dropout and learning rate optimization.

For model training and testing purposes, the data set is randomly split at the ratio of training: validate: test = 7:2:1. The structures and parameters of MLP, CNN and bLSTM are optimized based on a series of trials. There are a number of experiments that have been implemented to explore optimal hyperparameters, including dropout rate, learning rate, and network depth. Figure 10a presents that the best dropout rates for MLP and bLSTM are 0.2 while for CNN is 0.5. Learning rate controls how much the weights in neural networks are adjusted with respect to the loss gradient [55,56]. To explore a better initial learning rate in the decaying scheme, experiments are implemented. Figure 10b indicates the initial learning rate of $1\times 10^{-3}$ suits for all the three methods.

Different depths of these networks are also tried while other parameters remain the same. More layers lead to a decrease of prediction or overfitting whereas fewer layers may not be sufficiently trained. Figure 7, Figure 8 and Figure 9 provide more details of the final topologies with the suitable depth.

### 4.2. Decoding Results

The features are trained, validated and tested by MLP, CNN and bLSTM, respectively. The key hyperparameters of the deep learning models are displayed in Table 2. The same initial learning rate, activation function and batch size are used in the three decoders, while different optimizers and dropout rates are applied.

MLP, CNN and bLSTM are implemented in Keras (on top of TensorFlow), which offers many flexible functional APIs to build and optimize deep learning structures and parameters. Batch normalization is applied for all models to obtain smaller training and validation loss. In particular, the function ‘ReduceLROnPlateau’ is called to reduce learning rate, with factor = 0.2, patience = 20 and min_lr = 0.5$\times 10^{-6}$. In early stopping, patience is set to 80, which means training is stopped if the loss does not decrease after 80 epochs.

**Figure 11:**
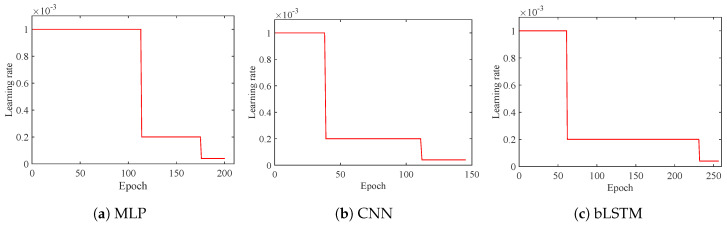
Learning rate of prediction in decoders.

Figure 11 shows the learning rate changes along with epochs during the training. All the three models are initialized with the same learning rate which is then decayed in different epochs. bLSTM takes more than 250 epochs in model training, while that of MLP is smaller and CNN consumes the least number of epochs.

**Figure 12:**
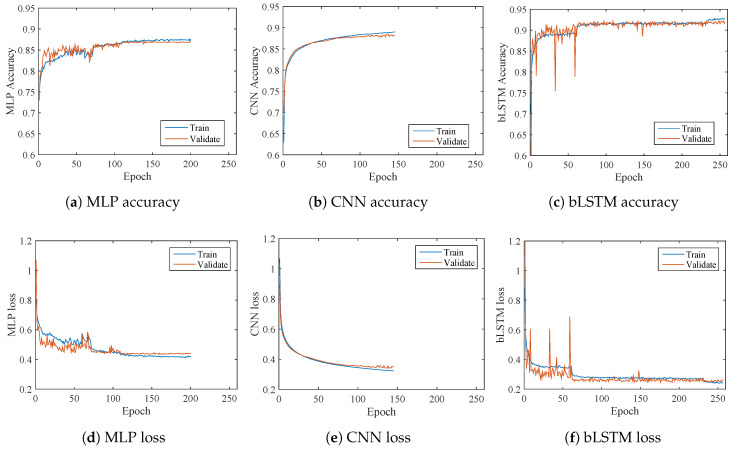
Training profile on the feature set by three deep learning models. (**a**) and (**d**): training on MLP. (**b**) and (**e**): training on CNN. (**c**) and (**f**): training on bLSTM. Both training and validation results are shown in the above sub-figures.

Training profiles are provided in Figure 12. Figure 12a,d give the training details of MLP, where the accuracy becomes stable around 150 epochs and the validation loss stays about 0.45. In Figure 12b,e, CNN training achieves a little better validation results than MLP but a large number of epochs is required. bLSTM shows the best validation accuracy of 0.92 in Figure 12c and the lowest validation loss of 0.26 in Figure 12f, however, its computational efficiency is not as good as those of MLP and CNN since bLSTM needs a large number of epochs to complete the training. The validation performance lines generally follow the training processes, which means the models are generally well-trained without obvious overfitting or underfitting.

The accuracy of MLP, CNN and bLSTM on the test set is 0.85, 0.87 and 0.90, respectively. Both training and test results indicate that bLSTM achieves the best performance among the three methods, though it takes a longer time to train.

**Figure 13:**
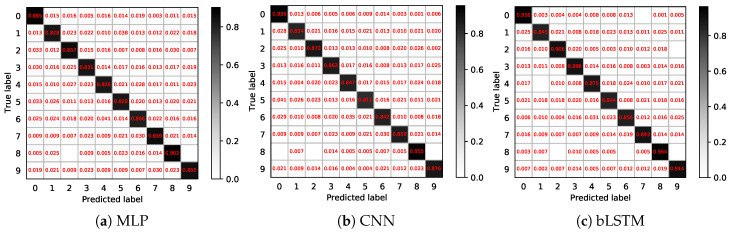
Confusion matrices of the three decoders.

The confusion matrix is computed to show more prediction details on the test set, as is shown in Figure 13. Labels 0 and 8 achieve the highest accuracy in all test predictions while labels 1, 5 and 6 have relatively low accuracy. Except for label 5, the accuracy of all others increases from Figure 13a,c. Samples are more likely to be classified as labels 0 or 8. In addition, all three decoders have an equal difficulty in distinguishing label 4 and label 6. This may be caused by similar neuromuscular activities.

## 5. Discussion

The valid samples for each label are various, due to (1) the impedance between an electrode and skin surface always changes in different experiments, even for the same participant; (2) the inherent differences in the speech intention of participants; and (3) the different responses from the neuromuscular activities to individual words in silent speech recording. The data set, as shown in Table 1, is still acceptable though small label imbalance exists, because each label is fully trained. The impedance reduction and preprocessing algorithm optimization will be further studied to increase the rate of valid samples.

MLP, CNN and bLSTM are trained and applied to decode the sEMG on the same platform (Intel i5-7400 CPU @ 3 GHz). bLSTM obtains highest accuracy around 0.92, with largest time consumption of almost 10 h. For CNN, the performance is not as good as bLSTM (0.88), but it consumes the least time (6 h) for proper model training. Though MLP takes less time (8 h) than bLSTM, its accuracy of 0.87 is worst among the three models. The bi-directional structure in bLSTM can generate better decoding results than MLP and CNN. Therefore, bLSTM suits the silent speech recognition if time consumption is less important. In test experiments, it takes no more than 50 ms to predict a new sEMG sample for the three models, which means instant prediction can be obtained to satisfy a real-time system.

The technology of silent speech decoding can be output in two forms, text code and synthetic speech [9,57]. It is up to the practical requirements. The speech pattern only appears in sEMG form regardless of audible or silent speech, so the privacy is ensured by the subject.

Silent speech decoding investigated in this paper is promising in possible applications: medical prostheses that help people with speech disabilities; hands-free peripheral device control; communication in privacy or noisy ambience [22,57,58,59]. The accuracy of single word is not high enough for piratical use. Communication also requires more complicated expression than the single words. Semantic dependency may help silent speech recognition in such potential applications, so phrases or even sentences may need to be researched.

Currently, 10 electrodes (2 for ground, 2 pairs of bipolar and 4 monopolar electrodes) for 6 channels are needed, and an integrated electrode array will be developed to improve the wearability. Furthermore, the electrode positions and channel number might be optimized to improve the performance and simplify the data collecting device. Online learning is another possible future research, as it is useful in data augmentation.

## 6. Conclusions

In this paper, it is demonstrated that spectrogram features combined with deep learning models can be applied to the silent speech decoding task, where bLSTM outperforms other methods. Result analysis indicates that synergic information hidden in multi-channel sEMG can provide useful features for recognition. It is suggested that the synergic exploration in silent speech decoding should be extended to phrases or even sentences, not only limited to a single word. 

## Figures and Tables

**Table 1:** Valid samples.

Label	‘0’	‘1’	‘2’	‘3’	‘4’	‘5’	‘6’	‘7’	‘8’	‘9’
Word	’噪’	’1#’	’2#’	’ 前’	’后’	’左’	’右’	’快’	’慢’	’停’
Samples	7964	6707	6814	6978	6593	6510	6682	6883	7614	6524

**Table 2:** Hyperparameters.

Model	Optimizer	Dropout	Learning Rate	Activation	Batch Size
MLP	adam	0.2	1$\times 10^{-3}$	ReLU	32
CNN	adadelta	0.5	1$\times 10^{-3}$	ReLU	32
bLSTM	rmsprop	0.2	1$\times 10^{-3}$	ReLU	32
